# The Healing Effects of Aquatic Activities and Allogenic Injection of Platelet-Rich Plasma (PRP) on Injuries of Achilles Tendon in Experimental Rat

**Published:** 2015-01

**Authors:** Hamid Rajabi, Homa Sheikhani Shahin, Manijeh Norouzian, Davood Mehrabani, Seifollah Dehghani Nazhvani

**Affiliations:** 1 Department of Exercise Physiology, Faculty of Physical Education and Sport Sciences, Kharazmei University, Tehran, Iran;; 2 Stem Cell and Transgenic Technology Research Center, Department of Pathology, Shiraz University of Medical Sciences, Shiraz, Iran;; 3 Department of Veterinary Surgery, School of Veterinary Medicine, Shiraz University, Shiraz, Iran

**Keywords:** Aquatic activities, Platelet Rich Plasma, Healing, Tendon, Rat

## Abstract

**BACKGROUND:**

Clinical tendon injuries represent serious and unresolved issues of the case on how the injured tendons could be improved based on natural structure and mechanical strength. The aim of this studies the effect of aquatic activities and alogenic platelet rich plasma (PRP) injection in healing Achilles tendons of rats.

**METHODS:**

Forty rats were randomly divided into 5 equal groups. Seventy two hours after a crush lesion on Achilles tendon, group 1 underwent aquatic activity for 8 weeks (five sessions per week), group 2 received intra-articular PRP (1 ml), group 3 had aquatic activity together with injection PRP injection after an experimental tendon injury, group 4 did not receive any treatment after tendon injury and the control group with no tendon injuries. of 32 rats. After 8 weeks, the animals were sacrificed and the tendons were transferred in 10% formalin for histological evaluation.

**RESULTS:**

There was a significant increase in number of fibroblast and cellular density, and collagen deposition in group 3 comparing to other groups denoting to an effective healing in injured tendons. However, there was no significant difference among the studied groups based on their tendons diameter.

**CONCLUSION:**

Based on our findings on the number of fibroblast, cellular density, collagen deposition, and tendon diameter, it was shown that aquatic activity together with PRP injection was the therapeutic measure of choice enhance healing in tendon injuries that can open a window in treatment of damages to tendons.

## INTRODUCTION

Tendons are live tissues which respond to the mechanical loading by modifying the metabolism, the structure and the mechanical features that get adapted to these pressures. Due to the adaptations of physical training, the rate of cross-sectional area of the tendon, the tensile strength and tendon fibroblasts and also the production of type I collagen would increase. On the other hand, improper exercise may lead to tendon injuries.^1^

These injuries along with skeletal muscle injuries are called under an umbrella term of musculoskeletal soft tissue injuries and they could be categorized as overuse or acute injuries.^2^^-^5 The most significant issue after tendon injuries is the time-consuming process of healing and its adverse effects on tendons mechanical and chemical features. Some studies suggested that various operating pressures and controlled movements on tendons lead to an increase in tendon repair strength, especially in Achilles tendons. Also, it has been observed that the collagen density, tendons size and density of collagen fibers increased after exercise and these ultra-structural changes were revealed in tendons mechanical performance improvement. Researchers It was shown that swimming exercises if began immediately after the injury and during the peak of inflammation would lead to an increase in unnatural tissue strength.^3^

There is currently little information on tendon adaptation after an exercise, especially after the injuries. Most injuries during long-term exercises are limited to tendons due to repetitive movements; hence, understanding the way tendons adapt to injuries and the treatment measures are important.^4^ Therefore, applying new treatment techniques such as injection of PRP could be among the effecting procedures suggested for reaching this end.^5^

PRP is a bioactive feature of blood which has a higher concentration of platelets comparing to the typical baseline blood platelet and due to having one million or more platelet per cubic milliliter of blood, is of great clinical value.^6^ Platelets are enucleated blood particles derived from the fragmentation of megakaryocytes circulating in an inactivate form till they come into contact with endothelial damaged regions. They work via the degranulation of the α-granules in platelets, which contain the synthesized and pre-packed growth factors. The most potent ones in restoring damaged tissues are VEFG, TGFβ, PDGF, IGF and EGF.^7^

These growth factors would directly bind to the surface of cell membranes to activate hemostasis and healing. They induce internal cellular signaling that stimulates angiogenesis, cell proliferation, cell differentiation and new matrix formation in tissue repair. Autologous platelet-rich plasma (PRP) is produced simply as needed emphasizing its clinical importance.^7^

Chaudhary *et al.* (2012) studied PRP in treating inflammation in the epicondyle suggesting that the release and distribution of vascular tissues did not follow a coherent and unified pattern due to the lack of emergence of angiofibroblastic reaction.^8^ Valuable information were found on the effect of PRP on survival and regeneration of autologous cartilage grafts as the extent of angiogenesis and the diameters of vessels were more prominent in the side applying PRP.^9^ Modarresi (2013) demonstrated that addition of 20% PRP to fat grafts resulted into a better fat grafting survival, a less bruising and inflammation reaction, and easier application of fat grafts due to liquefaction effect of PRP.^7^ Lane *et al.* (2013) studied the application of PRP in increasing the tendons performance. They claimed that injecting PRP in rabbits with patellar tendon injury lead to reformation of collagens which had a positive effect on healing process.^10^

Considering the unique features of tendons which are reflected as a lazy and almost vessel-less structure with a low capacity in healing, novel treatment methods are highly required. By the advancements in science and research on growth factors in healing tissues, using PRP as a rich source of growth factors in sport and exercise medicine has increased and it is believed that the increase in density and concentration of these factors could be associated with platelet density and concentration. In fact, researchers believe that the higher the platelet density and concentration in a sample is, the healing procedures improves.^11^

Hence, considering the fact that acute injuries of the ligaments and tendons which are observed by age and participating in sports events and the impacts of these injuries on athletes’ life and performance, accelerating the healing procedure and their early return of athletes to the sports events are of great significance. On the other hand, due to the complexity of healing procedure in which many molecules are involved, growth factors play a key role in this, the coordinate increase of these growth factors after physical activities is considered as a stimulant for considerable increase in their reproduction capacity.^11^ PRP production is simple and with low cost in providing growth factors for healing of injured tissues.^7^ So this study determined the effect of aquatic activities and allogenic injection of PRP in healing of Achilles tendon injuries. 

## MATERIALS AND METHODS

Forty male Sprague-Dawley rats were provided from Laboratory Animal Center of Shiraz University of Medical Sciences, Shiraz, Iran and housed under controlled temperature (22°C) and lighting (12:12 light-to-dark ratio; light on at 7:30A.M.) conditions in transparent polycarbonate cages with (54×18×18 cm) dimensions for 3 months. They had access to food and water ad libitum. All procedures were done based on laws of Animal Care by Iran Veterinary Organization.

Forty male Sprague-Dawley rats weighing 200±20 g were randomly divided into 5 equal groups. Seventy two hours after a crush lesion formed on Achilles tendon using mosquito hemostat, group 1 underwent aquatic activity, group 2 received intra-articular PRP (1 ml), group 3 had aquatic activity together with PRP injection, group 4 just experienced tendon injury without any treatment and group 5 as the control group while did not undergo tendon injury and did not receive any treatment. 

In order to prepare allogenic PRP, initially, 3 Sprague Dawley rats were selected and anesthetized. Five milliliter of blood was provided from each rat and immediately transferred into tubes containing anticoagulant. Blood samples were centrifuged for 5 minutes at 2000 RPM so that three separate layers were formed based on density of blood components. The buffy coat layer was closely collected by a sampler and transferred into a micro-tube. The micro-tube was centrifuged for a second time for 5 minutes at 2000 RPM so that PRP portion was obtained from the surface. 

Aquatic activity was undertaken for 8 weeks (five sessions per a week). For adaptation, rats were put into a glass aquarium with the dimensions of 50×40×50 cm filled with 34°C water for 5 minutes for 3 days. Then the aquatic activity was extended for 8 weeks (5 days per week/5-30 minutes each session) (Table 1). After 8 weeks, the animals were euthanized and sacrificed and the Achilles tendon were removed and transferred into 10% formalin buffer for histological evaluation using hematoxylin and eosin (H&E) staining. The studied factors in histological evaluation were the number of fibroblasts, collagen deposition, cellular density and tendon diameter. It should be mentioned that the pathologist was blind to the samples. 

**Table 1:** Eight weeks incremental aquatic activity program of studied rats

**Week**	**Stage**
	**Introduction Stage:** In this stage, to have optional activity, rats were put into a glass aquarium with the dimensions of 50×40×50 cm filled with 34°C water for 5 minutes for 3 days.
1	**Overload Stage: **days 1 to 5: Aquatic activity: 5 min
2	days 1 to 5: Aquatic activity: 10 min
3	days 1 to 5: Aquatic activity: 15 min
4	days 1 to 5 : Aquatic activity: 20 min
5	days 1 to 5: Aquatic activity: 25 min
6	days 1 to 5: Aquatic activity: 25 min
7	days 1 to 5: Aquatic activity: 30 min
8	days 1 to 5: Aquatic activity: 30 min

Ethical approval for this project was obtained from Kharazmei University Ethics Committee. For statistical analysis, SPSS software (version 16, Chicago, IL, USA) was used applying Kolmogorov Smirnov, one-way ANOVA and Sheffeh post hoc tests. The significance level was considered a p value less than 0.05.

## RESULTS

**Fig. 1:**
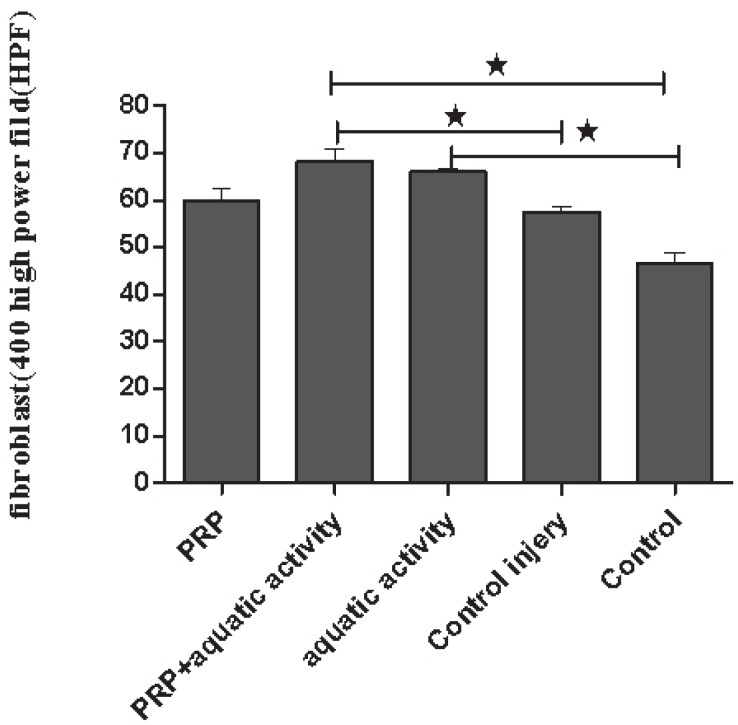
The mean of fibroblast numbers in different groups. *p<0.05

There was a significant increase in number of fibroblast (*p*=0.001), collagen deposition (*p*=0.001) and cellular density (*p*=0.001) in groups 2 and 3 in comparison to other groups. However, there was no significant difference among the studied groups based on their tendons diameter (*p*=0.4) (Table 2). Regarding just the number of fibroblasts, a significant increase was seen in group 3 in comparison to group 4 (*p*=0.04) and group 5 (*p*=0.001) and in group 1 comparing to group 5 (*p*=0.001) (Figure 1). 

**Table 2:** Mean and standard deviation of effective factors related to the healing of achilles tendon in studied groups.

**Variables**	**Fibroblasts**	**Collagen deposition**	**Cellularity**	**Tendon diameter**	**Total**
**Group**					
1: Aquatic activity	66±2.1	1±0.0	1.5±0.0	1780.8±379.1	69±2.1
2: PRP	59.7±6.9	1.4±0.5	1.7±0.4	1782.8±455.2	62.85±7.05
3: PRP+aquatic activity	68.1±7.1	1.4±0.5	1.5±0.5	1804.62±483.9	71.14±7.1
4: Tendon injury without treatment	57.6±2.3	0.9±0.0	1.3±0.0	1529.7±349.1	60±2.3
5: Control with no tendon injury	46.8±4.6	1±0.0	1±0.0	1478.6±195.5	50±4.6

**Fig. 2:**
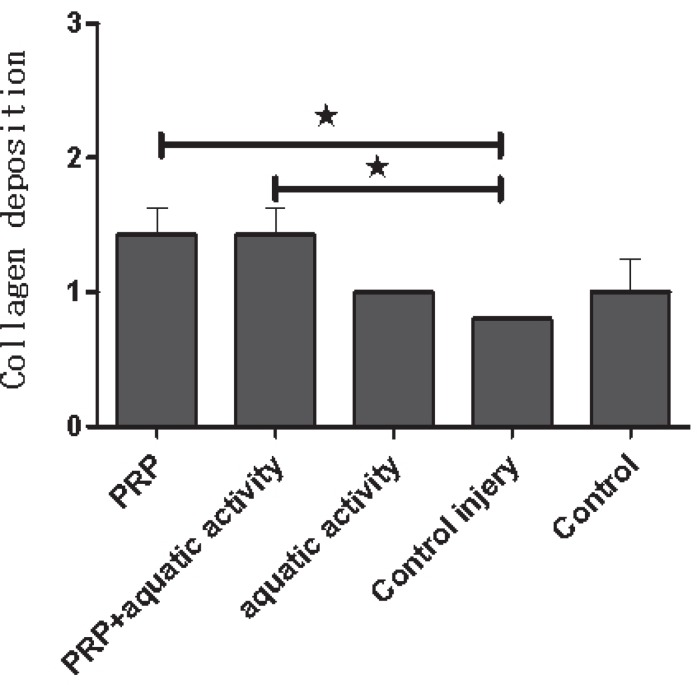
The mean of collagen deposition in different groups. *p<0.05.

**Fig. 3:**
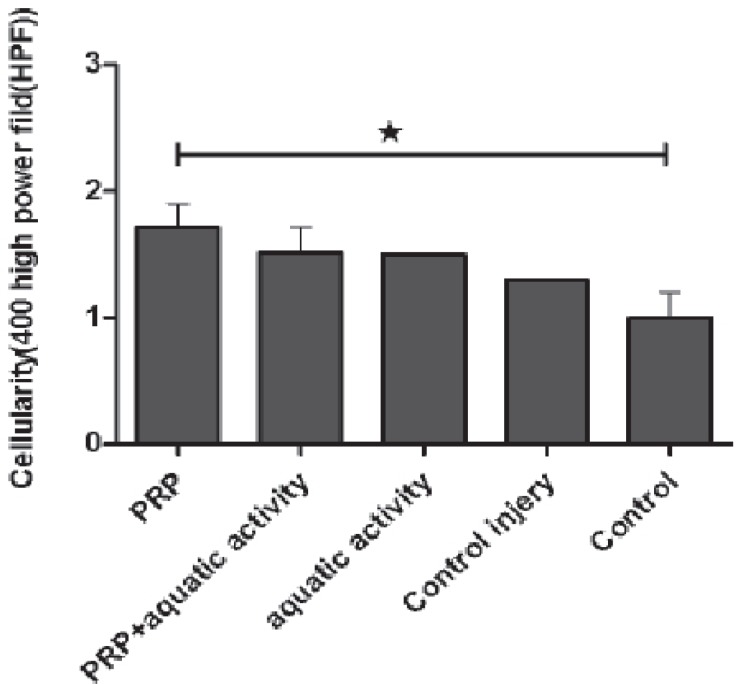
The mean of cellularity in different groups. *p<0.05.

A 42 and 38 percent healing increase in Achilles tendons were visible in groups 3 and 1, respectively. Regarding the collagen deposition, a significant increase was noticed in group 3 comparing to group 5 (*p*=0.001) and in group 2 comparing to group 4 (*p*=0.001) (Figure 2). There was a significant difference between group 1 and group 5 too (*p*=0.03) (Figure 3). Regarding tendon diameter affecting healing process, tendon diameter in PRP, aquatic activities and aquatic activities along with PRP groups showed an increase compared to the control group, but the difference was not statistically significant.

**Fig. 4:**
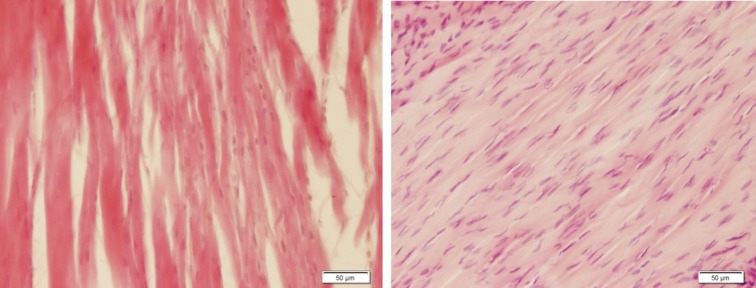
Tendon tissue (H&E, ×200). Left: Moderate healing in tendon revealing collagen deposition in the group undergoing aquatic activity. Right: High fibroblasts proliferation with collagen deposition in the group undergoing aquatic activity together with PRP injection

Figure 4 shows a moderate healing in Achilles tendon with collagen deposition in group 1 and a high fibroblast proliferation with collagen deposition in group 3. PRP injection along with aquatic activity and PRP injection alone could have a 55 percent increase in collagen deposition, comparing to the 4^th^ group and the difference was significant (Figure 4).

## DISCUSSION

Our findings revealed that aquatic activity together with PRP injection and aquatic activities alone could accelerate the healing process in injured Achilles tendon. In aquatic activity together with PRP injection and aquatic activity alone groups, there were 42 and 38 percent increase in healing of Achilles tendons, respectively, comparing to the control group. 

The benefit and safety of PRP were confirmed in more than 5,000 studies,^12^ regarding wound healing,^13^^-^^15^ tendon and cartilage healing,^16^^-^18 corneal healing^19^ and skin rejuvenation.^20^ PRP is also used more often in the plastic, reconstructive and aesthetic surgery fields.^21^ Mishra *et al.* (2006) applied PRP in healing of elbow tendons showing a significantly effective decrease in pain resulting to healing of tendons injuries.^22^

Kon *et al.* (2009) studied the application of PRP in healing of patellar tendon injuries in runners and jumpers. They observed a significant improvement in the pain, stiffness and strength of the tendons after six-months post-PRP injection.^23^ Also, Lopez *et al.* (2010) found that use of PRP could be considered as an effective treatment without any need for surgery in treating Achilles and plantar tendon injuries.^24^

Results from our study on application of PRP as a convenient treatment in accelerating tendon healing were in accordance with other studies even various methods were used for healing of Achilles tendon but PRP could significantly accelerate the tendons healing when it was used along with aquatic activity, so tendon healing may be accelerated by swimming. 

It was previously shown that swimming trainings if began immediately after the injury and at the peak of the inflammation, it would lead to an increase in tissue strength.^25^ Also, it was indicated that exercise could lead to 18-80 percent increase in the number of platelets and their size.^26^ Hence, PRP which contains platelets can lead into activation and accumulation of more platelets in the injured areas helping with the starting and acceleration of inflammatory responses by the host.^27^

In our research, the number of fibroblasts in the group of aquatic activity along with PRP injection increased when compared to the injury and control groups. Additionally, aquatic activities alone, lead to a 43 percent increase, comparing to the control group and these changes were statistically significant. Ernie (2010) and Graziani *et al.* (2005) showed that PRP could stimulate the fibroblasts proliferation and lead to an increase in their number.^28^^,^^29^ The results from this research on the increase in the number of fibroblasts are in accordance with aforementioned studies. 

Hence, considering the current results, it was noted that in response to injuries, fibroblasts would migrate to the injured areas and provide the secretion of collagen type III, which is further substituted by collagen type I.^27^ On the other hand, foreign bodies can lead to stimulation in the affected area and extend the inflammatory stage.^27^ Accordingly, the PRP injection could be considered as a convenient treatment in inflammatory reactions. Scientific and clinical studies suggested that application of PRP is an appropriate method in increasing the healing mechanisms and its accumulation may lead to an increase in the number of fibroblasts.^28^

Moreover, mechanical loading could impact collagen homeostasis and mechanical stimulations increasing the collagen presence in various tissues such as kidney, heart, arteries and respiratory system. Same results have been reported in vitro on tendons fibroblasts and ligaments after training and exercise in long-term periods.^30^ Therefore, it seems that mechanical simulations and exercises could increase the number of fibroblasts confirming the results for the current study that aquatic activities lead to an increase in the number of fibroblasts. 

Also, collagen deposition was another histological factor in healing procedure which was studied in our research. The results of our study suggested that PRP injection along with aquatic activity and PRP injection alone could have a 55 percent increase in collagen deposition, comparing to the injured group, and the difference was significant. De Mos *et al.* (2010) studied various amounts of PRP on human tenocyte and came to this conclusion that PRP could have a positive role on collagen production, cell proliferation and emergence of endogenous growth factors.^31^ Therefore, with an overview of the current results, the effective role of PRP in increasing the amount of collagen should be mentioned. It could be claimed that one of the clinical features of PRP is the release of bioactive proteins during the activation of platelets. The first release of proteins takes place 10 minutes after their activation and continues through platelets life span. These bioactive proteins include growth factors and cytokines released from platelets alpha particles. They could activate intracellular pathways and lead to the production of proteins needed for cellular proliferation, matrix formation and collagen combination.^7^

If aquatic activities has an impact on efficacy of PRP in collagen synthesis, hence, the importance of the role of aquatic activities on the rate of collagen synthesis and as a result tendons healing must be emphasized. In accordance with this issue, it could be said that collagens’ metabolism are affected by body activities; that is, a decrease in the activity may lead to a decrease in production of connective tissue. In contrast, exercise can increase the differentiation rate and connective tissue change in tendons. This is due to the physiological adaptation and injuries healing effects on extracellular matrix structure resulting from exercises.^32^

Furthermore, histopathological studies indicated that cellular density in PRP injected group was significantly different from the control group. In this regard, Lane *et al.* (2013) is their study used PRP to increase tendons cellular density and performance. They showed that collagen amount and cellular density increased by an increase in metabolic activity, which could have a positive impact on healing procedure.^10^ Results from current study were in accordance with aforementioned research. 

Tendon diameter was among the effective factors in healing process which was studied in this research. Our findings suggested that tendon diameter in PRP, aquatic activities and aquatic activities along with PRP groups had an increase compared to the control group. However, these changes were not significant. Michna *et al.* (1984) showed that training on treadmill for a week could increase the size of collagen fibers and tendon diameter in rats.^33^ In another research, Curwin *et al.* (1988) stated that intense endurance exercises lead to an increase in collagen deposition in Achilles tendon.^34^ Hence, with an overview of the available and current research results, it seems that the difference observed in this research could be due to the difference in type, intensity and duration if activities or type of participants. 

Thus, by an overview on results from current research and studying effective factors on tendon healing process, it could be reported that PRP use and aquatic activity could affect almost all activities engaged in healing trend such as accumulation of monocytes, and the fibroblasts proliferation leading to formation of matrix and collagen synthesis that can be beneficial in tendon healing. 

Cell proliferations in tendons occur by various chemical mediators such as growth factors, hormones, and cytokines. The main role of growth factors in increasing the cell survival and proliferation and as a result tissue rebuilding and healing is of great significance. Recent studies suggested that few successes have been gained using separate growth factors as treatment method. Direct injection of growth factors in injured tissues could lead to a temporary movement. Based on our findings on the number of fibroblast, cellular density, collagen deposition, and tendon diameter, it was shown that aquatic activity together with PRP injection was the therapeutic measure of choice enhances healing in tendon injuries that can open a window in treatment of damages to tendons. 

## Note

Please cite this paper as:

Rajabi H, Sheikhani Shahin H, Norouzian M, Mehrabani D, Dehghani Nazhvani S. The Healing Effects of Aquatic Activities and Allogenic Injection of Platelet-Rich Plasma (PRP) on Injuries of Achilles Tendon in Experimental Rat. World J Plast Surg 2015;4(1):66-73.
